# Cost-utility analysis of early reconstruction surgery versus conservative treatment for anterior cruciate ligament injury in a lower-middle income country

**DOI:** 10.1186/s12913-024-11212-8

**Published:** 2024-07-09

**Authors:** Romy Deviandri, Hugo C. van der Veen, Abdul Khairul R. Purba, Ajeng V. Icanervilia, Andri MT. Lubis, Inge van den Akker-Scheek, Maarten J. Postma

**Affiliations:** 1 Department of Orthopedics, University of Groningen, University Medical Center Groningen, Groningen, The Netherlands; 2 Department of Surgery-Faculty of Medicine, Universitas Riau, Arifin Achmad Hospital, Diponegoro street no 1, Pekanbaru, 28133 Indonesia; 3 Division of Orthopedics-Sports Injury, Fit Centre Institute, Pekanbaru, Indonesia; 4 Department of Pharmacology and Therapy, Faculty of Medicine, Universitas Airlangga, Surabaya, Indonesia; 5 Department of Health Sciences, University of Groningen, University Medical Center Groningen, Groningen, The Netherlands; 6 Department of Economics, Econometrics & Finance, Faculty of Economics & Business, University of Groningen, Groningen, The Netherlands; 7 Department of Pharmacology & Therapy, Universitas Airlangga, Surabaya, Indonesia; 8 Center of Excellence in Higher Education for Pharmaceutical Care Innovation, Universitas Padjadjaran, Bandung, Indonesia; 9 Department of Radiology, Faculty of Medicine, Public Health, and Nursing, Universitas Gadjah Mada, Yogyakarta, Indonesia; 10 Department of Orthopedics-Faculty of Medicine, Universitas Indonesia, Cipto Mangunkusumo Hospital, Jakarta, Indonesia

**Keywords:** ACL rupture, Rehabilitation, Reconstruction, Cost-effectiveness, LMIC

## Abstract

**Background:**

The ideal approach for treating anterior cruciate ligament (ACL) injury is still disputed. This study aimed to determine the more cost-effective strategy by comparing early ACL reconstruction (ACLR) surgery to conservative treatment (rehabilitation with optional delayed reconstruction) for ACL injury in a lower/middle-income country (LMIC), Indonesia.

**Methods:**

A decision tree model was constructed for cost-utility analysis of early ACLR versus conservative treatment. The transition probabilities between states were obtained from the literature review. Utilities were measured by the EQ-5D-3 L from a prospective cohort study in a local hospital. The costs were obtained from a previous study that elaborated on the burden and cost of ACLR in Indonesia. Effectiveness was expressed in quality-adjusted life years gained (QALYs). Principal outcome measure was the incremental cost-effectiveness ratios (ICER). Willingness-to-pay was set at US$12,876 — three times the Indonesian GDP per capita in 2021 — the currently accepted standard in Indonesia as suggested by the World Health Organization Choosing Interventions that are Cost-Effective criterion (WHO-CHOICE).

**Results:**

The early ACLR group showed an incremental gain of 0.05 QALYs over the conservative treatment group, with a higher overall cost to society of US$976. The ICER of ACLR surgery was US$19,524 per QALY, above the WTP threshold of US$12,876. The ICER was sensitive to cost of conservative treatment, cost of ACLR, and rate of cross-over to delayed ACLR numbers in the conservative treatment group. Using the WTP threshold of US$12,876, the probability of conservative treatment being preferred over early ACLR was 64%.

**Conclusions:**

Based on the current model, early ACLR surgery does not seem more cost-effective compared to conservative treatment for ACL injury patients in Indonesia. Because the result was sensitive to the rate of cross-over probabilities from the conservative treatment alone to delayed ACLR, a future study with a long-term perspective is needed to further elucidate its impact.

**Supplementary Information:**

The online version contains supplementary material available at 10.1186/s12913-024-11212-8.

## Introduction

The annual incidence of anterior cruciate ligament (ACL) injury, when adjusted for age and gender, ranges from 0.03 to 0.04% [1]. In Indonesia (population 274 million), the number is thought to be close to one million per year [2]. Orthopedic surgeons commonly undertake anterior cruciate ligament reconstruction (ACLR) surgery as treatment, with an estimated number rising globally, including Indonesia [3, 4]. Based on data from Indonesia’s three prominent ACL-implant companies, ACLR procedures increased by more than 50% from 2018 to 2022 (1575 implants in 2018 vs. 2481 in 2022). These numbers are expected to climb further due to the growing numbers of people that can afford to visit a health facility, plus the increased participation in sports activities [4].

Besides ACLR, ACL injury can be managed successfully without surgery in selected patients [5, 6]. Growing evidence shows no statistically or clinically relevant differences in clinical outcomes between early ACLR and conservative treatment (rehabilitation with optional delayed ACLR) [5, 7]. Kiadaliri et al. showed no economic benefit of early ACLR over conservative treatment for ACL injury [8]. Deviandri et al. specified conservative treatment may be a more cost-effective strategy in a general population with low and moderate activity levels [9]. Still, early ACLR is considered a more cost-effective strategy for ACL injury in athletes and young people with high activity levels. A recent clinical trial in England concluded that early ACLR was a management strategy superior to conservative treatment [10]. However, these results are from studies conducted in the USA and a European country, so these findings may not be transferable to a lower/middle-income country (LMIC) due to differences in healthcare systems, costs, and the standard routine care in each country. For example, Deviandri et al.(2022) showed that the mean cost of an ACLR in Indonesia was US$2,853, thus significantly lower than the costs of ACLR in other countries, such as the United States (US$24,707), the Netherlands (US$ 7,129), Sweden (US$ 5,760), Switzerland (US$ 7,391), Malaysia (US$ 4,354), and Thailand (US$ 5,710) [8, 11–15]. Therefore, studies in LMICs are needed to strengthen knowledge of cost-effectiveness in these areas, including Indonesia.

This study aims to determine the more cost-effective strategy by comparing early ACLR surgery to conservative treatment for ACL injury in LMICs, focusing on Indonesia as the fourth-most populous nation worldwide and the most populous in Southeast Asia. We hypothesized that early ACLR surgery is not more cost-effective than conservative treatment for ACL injury in Indonesia.

## Methods

### General model overview

We investigated the cost-utility of early ACLR compared to conservative treatment using a decision tree model. We developed a cost-effectiveness model estimated from the societal perspective. After obtaining Institutional Review Board (IRB) approval, data on outcome measures were derived from the prospective study cohort at a local hospital between 2021 and 2022. Data on cost was obtained from a previous study that elaborated on the burden and cost of ACLR in Indonesia [15]. The states of transition probabilities were extracted from the literature. To that end, the search from an electronic database search of PubMed, Medline, and Embase was conducted for relevant studies in the timeframe from the inception to October 2022. The key strings for the searching strategy were based on the PICO method (Patients, Intervention, Comparator, Outcomes) method (Supplementary, Table 1). A search was done using the Boolean operator “OR” inside sequences of terms with close or comparable meanings and “AND” for one or more sequences of terms that incorporated entirely different meanings. Search terms or phrases encoded in Medical Subject Headings (MeSH) were employed. Subsequently, the terms or phrases used in PubMed were translated to the Embase database for further search.

**Fig. 1:**
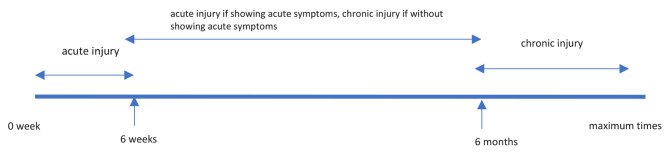
The time scale of acute and chronic injury. Chronic injury is defined as existing more than six months after the injury or less than six months without showing acute symptoms (i.e. inflammation, swelling, knee effusion)

**Fig. 2:**
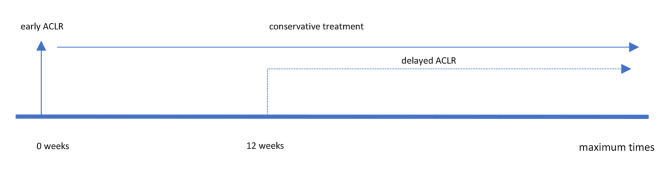
The time scale of early ACLR (vertical line), conservative treatment (horizontal line), and delayed ACLR (dash-line). Delayed ACLR is performed if needed when the conservative treatment fails after 12 weeks

Acute ACL injury was defined as existing ACL injury maximally six weeks old, or more than six weeks with showing acute symptoms (i.e. inflammation, swelling, knee effusion); chronic ACL injury was defined as existing at more than six months after the injury, or less than six months without showing acute symptoms (Fig. 1). Early ACLR treatment was defined as early surgical management (reconstruction) without prior conservative treatment, while conservative treatment was defined as non-surgical management by a structured rehabilitation program for a minimum of 12 weeks with the option of ACLR later only if needed (delayed ACLR) (Fig. 2).

Two studies showed that in Indonesia, almost all patients with an ACL injury came to the hospital clinician at a chronic stage, i.e. more than six months after the injury [16, 17]. Therefore, the literature review aimed for studies including patients with a chronic ACL injury representing the natural setting in Indonesia. The selected studies were deemed eligible if they evaluated the clinical benefits of early ACLR versus conservative treatment after chronic ACL injury and were designed as a randomized controlled trial.

The literature reviews initially identified a total of 1812 articles in the primary search. After screening and assessing for eligibility, we found only one eligible study — the ACL Surgery Necessity in Non-Acute Patients (ACL SNNAP) trial [10]. We thus used this study as a source of probabilities.

The cycle length for the model in this study was run for 18 months following the results of the literature review (ACL SNNAP). The model and its analysis were performed using a general decision analysis software package (TreeAge Pro Health Care 2022; TreeAge Software Inc., Williamstown, MA, USA). The primary effectiveness outcome was utility expressed in quality-adjusted life years (QALY). The cost was estimated in US$2020. Both costs and utilities were discounted at 3% to reflect their present value.

### Model structure

**Fig. 3:**
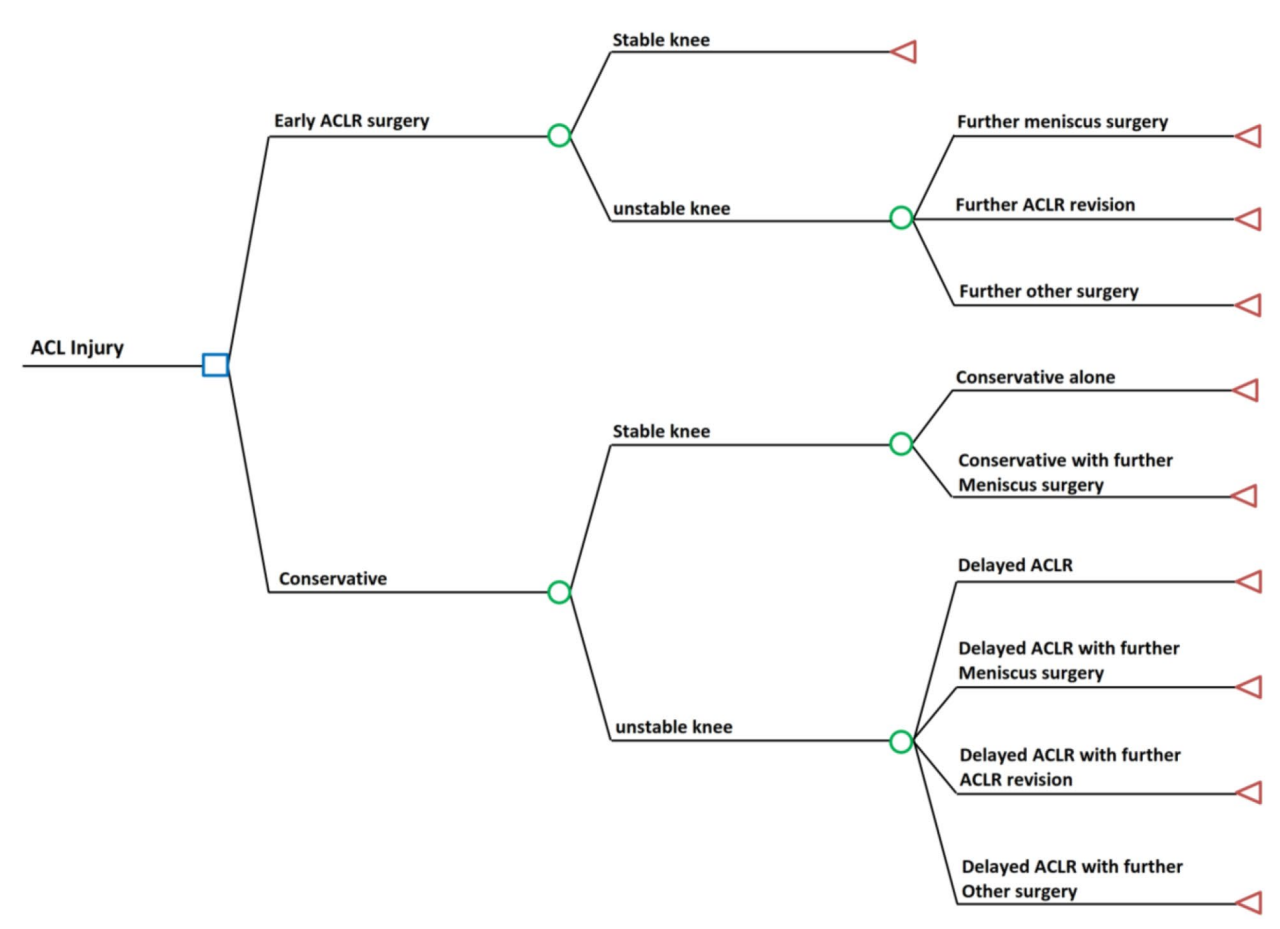
Decision tree model of ACL injury treatment between early ACLR and conservative treatment

>The decision tree model consisted of two primary interventions (early ACLR and conservative treatment). Patients in the early ACLR group entered a post-procedure state for 18 months, while those in the conservative treatment group entered an initial rehabilitation state for 18 months. All patients were assumed to have a stable knee at the end of the cycle of our decision tree model. Patients in the early ACLR arm could undergo additional surgeries consisting of (1) meniscus surgery – meniscectomy or meniscus repair, (2) ACL revision, or (3) other probable subsequent surgeries (other surgery) consisting of manipulation under anesthesia, removal of hardware, or basic knee arthroscopy. These surgery rates were taken from the ACL SNNAP study [10]. The probability rate of ‘other surgery’ in the ACLR group was additionally based on the authors’ clinical experience in practice as these were not found in the ACL SNNAP study. However, these options exist in daily practice and have been described widely in some literature [19, 24, 27–29]. In the conservative treatment group, patients could undergo arthroscopy without ACLR for meniscus treatment. After that, patients were expected to have stable knees with conservative treatment alone. In case of persisting symptomatic instability, a delayed ACLR could be performed (Fig. 3). The re-operation rates were taken from the ACL SNNAP study (Supplementary, Table 3) [10].

### Model parameters

#### Utilities

Utility values range from 0,0 (worst health state) to 1.0 (perfect health state). Utilities in this model were derived from the EQ-5D-3 L values taken from the prospective cohort study in the authors’ local hospital. A clinically stable knee ascertained after early ACLR and conservative treatment was 0.86 and 0.81, respectively. The baseline of utility value was 0.61 — the same for both groups. All of the utility values are summarized in Supplementary, Table 2.

#### Procedure rates

The rate of unstable knees from the ACL SNNAP study was used in our model to estimate the number of patients undergoing rehabilitation that were stable or unstable. The rate of delayed ACLR in the conservative treatment group was 41% for 18 months. The specific number of visits for postoperative and nonoperative rehabilitation therapy were taken from the direct observation of the author at the local hospital as a daily practice in Indonesia — two visits per week for 36 weeks were used for postoperative rehabilitation therapy after ACLR. All procedure rates and model variables input data are summarized in Supplementary, Table 3.

#### Costs

The costs were calculated from a societal perspective as suggested by the International Society for Pharmacoeconomics and Outcomes Research (ISPOR) guidelines [18]. Direct costs were estimated using billing information that comprised the cost of beds, pharmacies, implants, surgeries, other fees, and overall costs. Next, the total payment was calculated by adding the outpatient facility reimbursement and the professional expenses for each service. The direct cost was obtained from a previous study [15]. The 2019 currency exchange rate (US$ 1 = 14,147,671 IDR) was used, as applied by the Organization for Economic Cooperation and Development (OECD), to convert Indonesian Rupiahs (IDR) into US Dollars (US$) [15]. We calculated the indirect costs from loss of salary and cost of transportation. These costs were based on a survey of the 50 patients of the author (RD) at a local hospital in Indonesia in 2022. All of the indirect costs are summarized in Supplementary, Table 2.

### Cost-utility analysis

The cost-utility analysis for each treatment approach was performed using the decision tree cohort model. Each method’s average costs, efficacy (QALYs), and cost-effectiveness (C/E) ratios were used as outcome measures. A quality-adjusted life year (QALY) is an outcome measure equal to the projected increases in life expectancy associated with a treatment choice, adjusted downward for any quality-of-life constraints. The incremental cost-effectiveness ratio (ICER), which is the ratio between the difference in costs and the difference in QALY of each strategy, is used to compute the primary outcome measurement by way of: ICER = (Cost early ACLR − Cost rehabilitation) / (QALY early ACLR − QALY rehabilitation) [19].

A specific intervention is deemed as highly cost-effective by the World Health Organization Choosing Interventions that are Cost-Effective criterion (WHO-CHOICE) when the ICER is lower than the willingness-to-pay (WTP), which is determined by the Indonesian cost-effectiveness threshold of gross domestic product (GDP) per capita. The intervention is cost-effective if the ICER is lower than three times the GDP per capita [20, 21]. A prior study conducted in Indonesia also used this approach [22]. We used the 2021 Indonesian GDP per capita of US$4,292 and the WTP criterion of US$12,876 to determine this threshold [23]. In this cost-effectiveness analysis, the preferred treatment strategy is considered more effective if ICER < WTP.

On each variable in the model, one-way, two-way, and tornado sensitivity analyses were run. Model findings are continuously calculated using changed versions in a sensitivity analysis. The output’s value ranges indicate how sensitive a result is to the model’s underlying assumptions. The variables classified as “sensitive” were those that, when changed across an acceptable range, also altered the chosen method. The variable was said to be “robust” if the preferred strategy remained constant. Monte Carlo analysis was utilized in conjunction with microsimulation and probabilistic sensitivity analysis for 5000 samples to create 95% confidence intervals for the results.

## Results

For a chronic ACL injury patient in Indonesia with over 18 months’ follow-up, the strategy of ACLR surgery would be estimated to cost US$4,266. Therefore, the ICER of ACLR surgery was US$19,524 per QALY, above the WTP threshold of US$12,876 (Table 1).

**Fig. 4:**
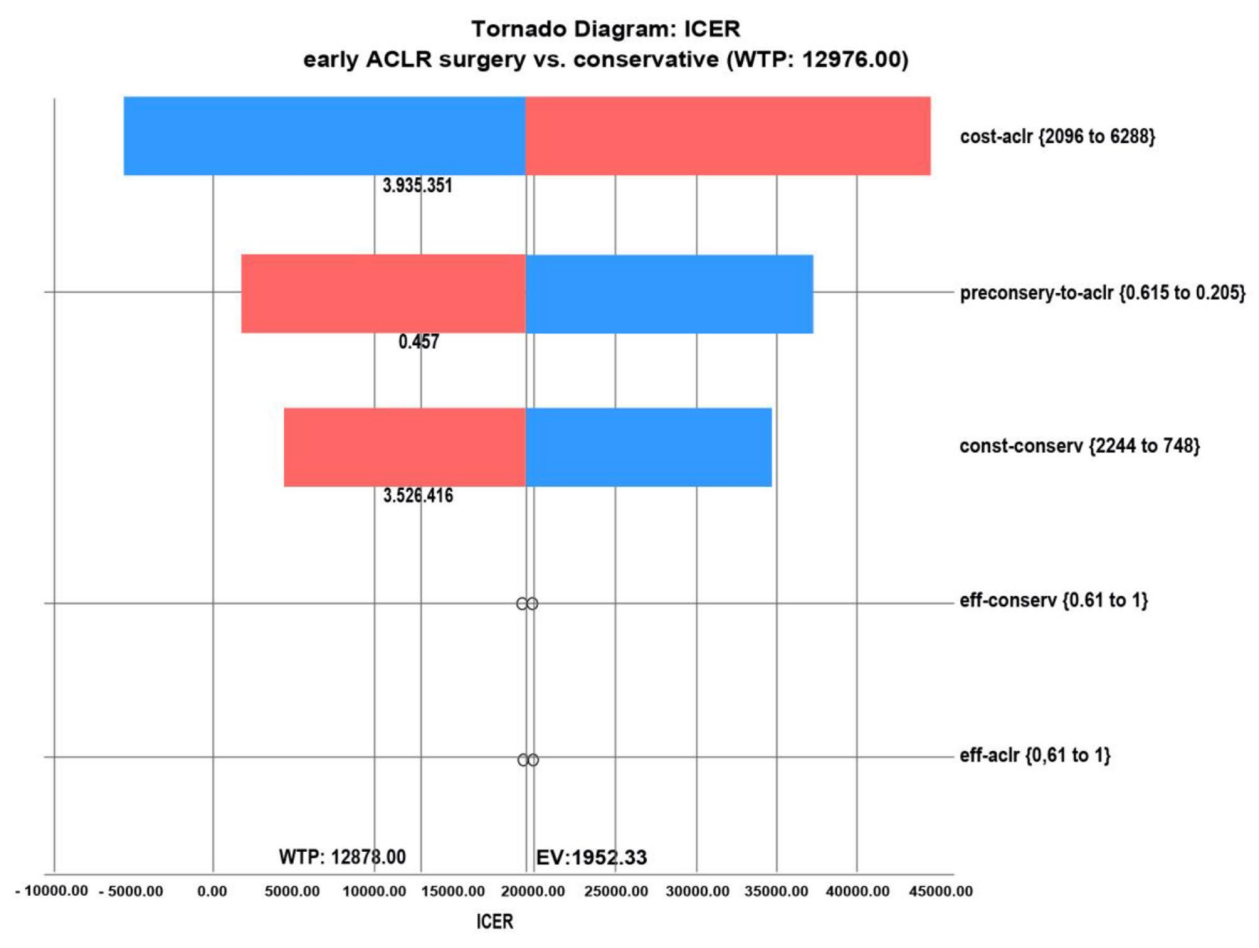
The tornado sensitivity analysis results between early ACLR and conservative treatment showed that ICER was sensitive to cost of ACLR (cost-aclr), cost of conservative treatment (const-conserv), and probability of delayed ACLR numbers in the conservative treatment group (preconserv-to-aclr). ICER was insensitive to quality of life after both treatment strategies (eff-conserv, eff-aclr). ICER, incremental cost-effectiveness ratio. WTP, willingness-to-pay. EV, expected value

**Fig. 5:**
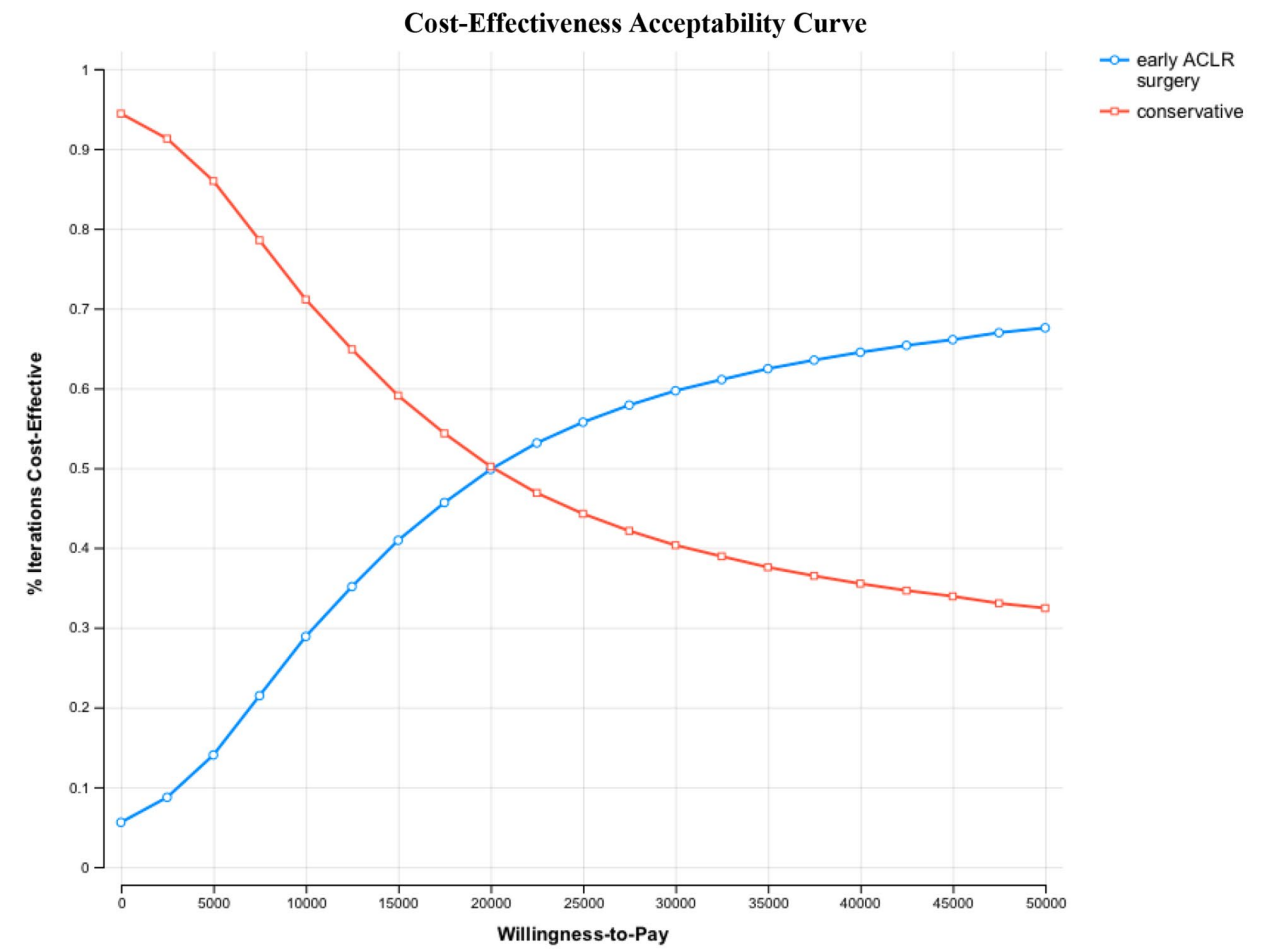
Acceptability curve of early ACLR and conservative treatment over a range of willingness-to-pay (WTP) thresholds. The probability of conservative treatment being cost-effective over early ACLR was 64% by using a WTP threshold of US$12,876

**Fig. 6:**
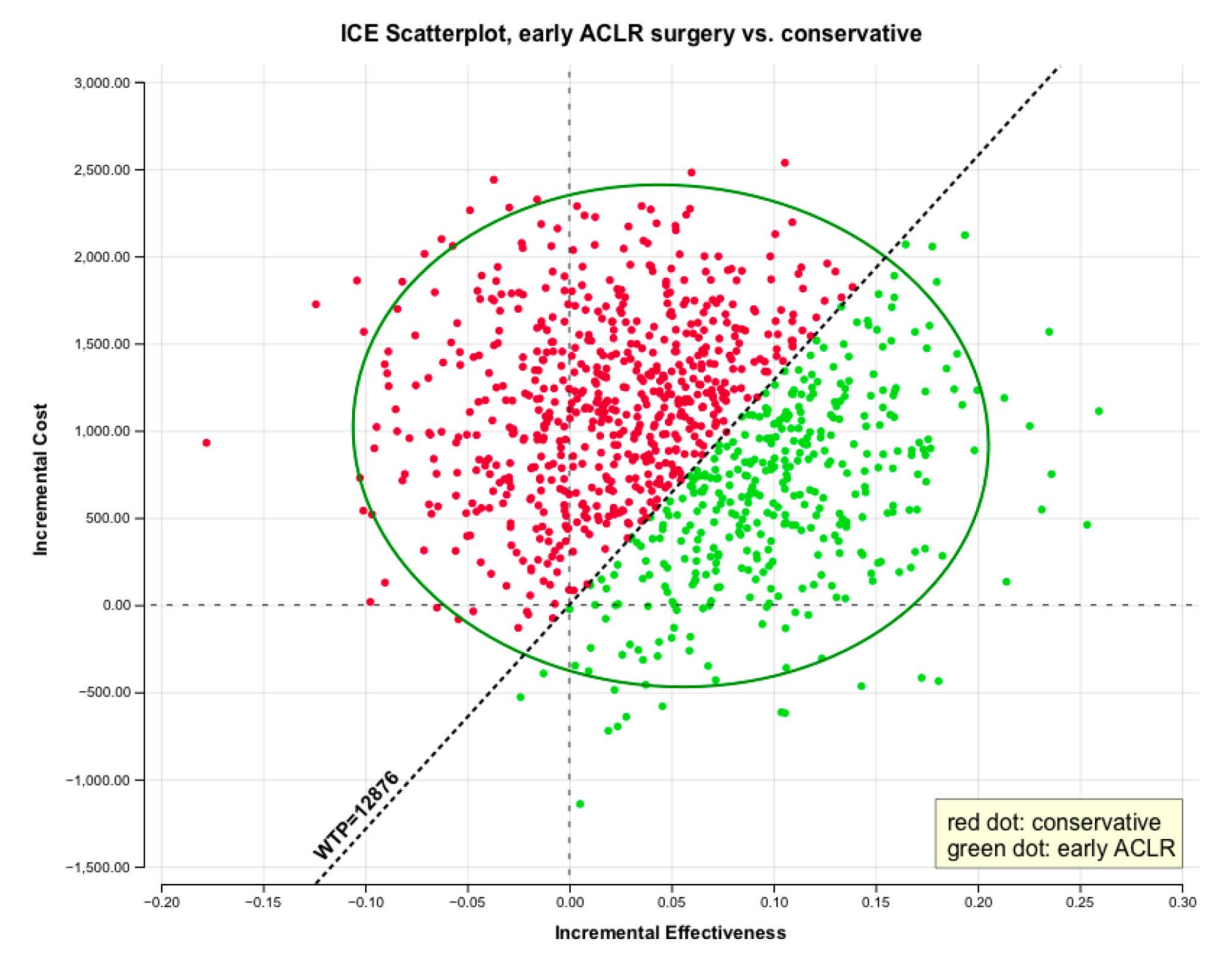
Monte Carlo sensitivity analysis of early ACLR compared with conservative treatment. The scatterplot depicts the incremental cost-effectiveness ratios derived from the Monte Carlo (probabilistic) sensitivity analysis for the first 1000 samples of the total 5,000 random samples. In a graph in the cost-effectiveness space, the incremental effectiveness is plotted on the x-axis, the incremental cost is plotted on the y-axis. Values in the upper right quadrant represent a strategy that is more costly but also more effective than the comparator, while in the lower right it is more effective and less costly than the comparator. Values in the upper left are more costly and less effective, and values in the lower left are less costly and less effective than the comparator

The ICER was sensitive to cost of ACLR, cost of the conservative treatment, and probability of delayed ACLR numbers in the conservative treatment group. The ICER was robust to QOL after treatment with both strategies (Fig. 4). The acceptability curve showed the proportion of samples comparing early ACLR to conservative treatment in various ranges of the WTP threshold. Using the WTP threshold of US$12,876, the probability of conservative treatment being preferred over early ACLR was 64%. However, the probability of early ACLR being preferred increased as the WTP threshold rose to 50% at US$20,000 of the WTP threshold and continued to rise after that (Fig. 5). The incremental cost and effectiveness of early ACLR compared to conservative treatment from 5000 samples is depicted in Fig. 6. The ellipse image shows the 95% confidence interval for differences between each strategy.

**Table 1:** Cost-effectiveness results between early anterior cruciate ligament reconstruction (ACLR) and conservative treatment

Strategy	Cost (US$)	Incremental cost (US$)	Quality-adjusted life years	Incremental effectiveness	Incremental cost-effectiveness ratio	Net monetary benefit (US$)
Conservative treatment	3,290	--	0.81	--	--	7,139
Early ACLR Surgery	4,266	976	0.86	0.05	19,524	6,806

## Discussion

This study aimed to determine the more cost-effective strategy by comparing early ACLR to conservative treatment for ACL injury patients in a LMIC, focusing on Indonesia. The results confirmed our hypothesis and showed that early ACLR surgery was not more cost-effective than conservative treatment for chronic ACL injury patients in Indonesia.

Many studies have shown ACLR surgery to be more cost-effective than conservative treatment [19, 24–26]. However, all those studies were conducted in developed countries, and mostly in acute ACL injury settings. It would therefore be questionable to adopt those results in LMICs, which have different costs for healthcare services and different modalities of usual care. This study used the local characteristics of Indonesia as representative of LMICs, showing indeed a different outcome as compared to the US and European countries. We found that ACLR surgery was not more cost-effective than conservative treatment for the chronic ACL injury setting, with an ICER of US$19,524 per QALY. The threshold of WTP in LMICs is lower than in developed countries. LMICs have a lower ability to pay for ACLR surgical treatment, making early ACLR less cost-effective than conservative treatment. However, early ACLR is still more effective than conservative treatment, with a number of utilities at 0.86 and 0.81, respectively.

The results of this study indicate 64% confidence of acceptability at the threshold of a WTP under US$12,876 per QALY gained by using Monte Carlo probabilistic sensitivity analysis. Using the tornado sensitivity analysis, the ICER was sensitive to cost of ACLR, cost of conservative treatment, and probability of delayed ACLR numbers in the conservative treatment group. If the cost of ACLR were lower than US$3,635.15, or the cost of conservative exceeded US$1,828.42, this would change the outcome and result in a situation where early ACLR becomes a more cost-effective strategy than conservative treatment. Also, if the cross-over probability from conservative alone to delayed ACLR exceeded 50%, conservative treatment would not be a more cost-effective strategy than early ACLR. The results of the ACL SNNAP study showed that the probability of conversion into delayed ACLR in the conservative treatment group was 41% in 18 months [10]. However, with a longer follow-up time, this percentage might be higher. Other studies also describe this phenomenon, showing an increase of over 50% from two to five years’ follow-up [27, 28]. The Knee Anterior Cruciate Ligament Nonsurgical vs. Surgical Treatment (KANON) study showed that cross-over probability from conservative treatment alone to a delayed ACLR increased by 51% after five years of follow-up [27]. Thus, based on the probability in this KANON study, it could be assumed that the early ACLR strategy could be preferred over conservative treatment from a longer-term perspective. Therefore, although the results of our study encourage clinicians and young active adult patients to consider conservative treatment as a primary treatment option, the clinician should be aware of the rate of delayed ACLR and pay more attention to the quality of the conservative treatment itself. A growing body of knowledge recommends a well-structured rehabilitation program for a minimum of 24 weeks to achieve a good result of a rehabilitative program [27–30].

In this study, ICER was robust to quality of life (QoL) after early ACLR and conservative treatment. Growing evidence shows no significant differences in clinical outcomes of QoL between early ACLR and conservative treatment [5, 10, 28, 29]. The slight discrepancy in clinical outcome between the two treatments could be the reason why, after both early ACLR and conservative treatment, QoL did not affect the ICER. In this study, we mainly collected data on QoL after ACL injury treatment from patients in the general Indonesian population. Besides, by using the data of the ACL SNNAP trial as an internal validation, the same mean difference of utility values was shown. We also used the utility values’ data of the SNNAP trial as a lower value threshold for conducting the probability analysis of a different number of utility values. The outcome of a probabilistic sensitivity analysis is now the suggested method for analyzing the effects of parameter uncertainty on the results of a cost-effectiveness analysis [31]. The result of the one-way sensitivity analysis showed that if the treatment of early ACLR could result in higher QoL in a specific population, such as athletes, as described by Stewart et al. [25] — with the threshold of 0.89 in this study — early ACLR could be a more cost-effective strategy than conservative treatment (Supplementary, Fig. 1). Furthermore, a previous study showed that early ACLR led to improved clinically compared with conservative treatment [10, 29].

This study has some limitations. First, we conducted this study from a short-term perspective, as long-term data on effectiveness and costs are currently lacking. However, according to ISPOR guidelines, a long-term perspective is preferred in health economic analyses [20]. Further research from a long-term perspective is needed to describe in more detail the cost-effectiveness strategy for ACL injury patients in LMICs. It could be hypothesized that the results change if the cross-over probability from the conservative treatment group to the early ACLR group increases in the long term by more than 50%, as shown by other RCTs in the acute ACL injury setting [27–29]. The Knee Anterior Cruciate Ligament Nonsurgical vs. Surgical Treatment (KANON) study showed that the probability of cross-over from conservative treatment alone to a delayed ACLR increased from two to five years’ follow-up [27, 28]. Based on the probability in this KANON study, from a long-term perspective the early ACLR strategy could be preferred over conservative treatment.

Next, we used data on QoL of ACL injury patients from an observational study with a limited sample size. Also, the information about costs was limited, especially for indirect costs, where we could only use data on loss of salary and transportation costs. Other indirect costs, such as productivity, should also be considered. Furthermore, the indirect cost data was used in this study only based on the survey from one local hospital in Indonesia. More data from other hospitals in Indonesia would provide a more complete overview of the costs for the whole of Indonesia. Future studies are needed to gain these data to increase the accuracy of our cost-effectiveness modelling. However, the results of this study still can be considered to adequately represent Indonesia’s setting. The sensitivity analysis showed that the ICER was insensitive to the indirect cost itself (supplementary, Table 3).

This is the first study to describe the cost-effectiveness strategy for chronic ACL injury patients in a LMIC. The study shows that an early ACLR strategy is not more cost-effective than conservative treatment for ACL injury patients in a LMIC, in contrast to many developed countries [19, 24–26]. It also provides a decision tree model for future cost-effectiveness studies in ACL injury patients, especially chronic ACL injury cases. The results of this study are helpful for clinicians, payers, health technology assessment organizations, regulators, and clinical guideline developers to decide on a preferred strategy for a healthcare program, particularly for ACL injury cases in LMIC. This study provided a management strategy for ACL injury treatment in LMICs, focusing on health economics in Indonesia. Although more research is needed, findings from this study do have important clinical implications. Clinicians in LMICs, particularly in Indonesia, are advised to choose the conservative treatment strategy if needed followed by a delayed reconstruction surgery, in patients with ACL injury in the general population. Furthermore, besides being more cost-effective, it also alleviates the problem of available surgical capacity in a particular region.

## Conclusion

Early ACLR surgery does not seem more cost-effective than conservative treatment for chronic ACL injury patients in LMICs, particularly in Indonesia. Long-term data on the rate of cross-over probabilities from conservative treatment alone to a delayed ACLR is needed to further increase the accuracy of our model.

### Electronic supplementary material

Below is the link to the electronic supplementary material.

Supplementary Material 1

## Data Availability

Data is provided within the manuscript or supplementary information files.
