# Supplementary material for: Cost-utility analysis of early reconstruction surgery versus conservative treatment for anterior cruciate ligament injury in a lower-middle income country
Source: BMC Health Serv Res. 2024 Jul 9;24:784. doi: 10.1186/s12913-024-11212-8 (PMC11234700; doi:10.1186/s12913-024-11212-8)
Supplement: Supplementary file 1 — Supplementary Material 1 [file 12913_2024_11212_MOESM1_ESM.docx]

**Supplementary**

Supplementary Table 1. PICO strategy and search terms or phrases encoded in Medical Subject Headings (MeSH) were used in PubMed

| PICO strategy and search terms |
| --- |
| P: anterior cruciate ligament injury  I: anterior cruciate ligament reconstruction  C: rehabilitation  O: treatment outcome  ("Anterior Cruciate Ligament Injuries"[Mesh] OR "Anterior Cruciate Ligament"[Mesh] OR ACL[tiab])  AND  ("Anterior Cruciate Ligament Reconstruction"[Mesh] OR "Reconstructive Surgical Procedures"[Mesh])  AND  ("Physical and Rehabilitation Medicine"[Mesh] OR "rehabilitation" [Subheading] OR "Rehabilitation"[Mesh] OR "Orthopedic Procedures"[Mesh])  AND  ("treatment outcome"[Mesh] OR Treatment Outcome[tiab]) |

Supplementary Table 2. The utility values and the indirect costs between ACLR and the conservative treatment group

| Group | Mean age | Baseline utility value | Utility value after treatment | Loss of salary per month | Transportation cost per meeting |
| --- | --- | --- | --- | --- | --- |
| Early ACLR | 27 ± 6.9 | 0.61 | 0.86 | 50 US$ | 3.3 US$ |
| Conservative treatment | 26 ± 6.0 | 0.61 | 0.81 | 50 US$ | 3.3 US$ |

Supplementary Table 3. Model Variables Input Data and Distribution for Sensitivity Analysis

| Variable | Base case value | Low value | High value | Distribution |
| --- | --- | --- | --- | --- |
| **Probability^#^** |  |  |  |  |
| Stable knee after ACLR | 0,977 | 0.488 | 1 | Beta |
| Unstable knee after ACLR | 0.023 | 0.012 | 0.034 | Beta |
| Stable knee after conservative treatment | 0.590 | 0.295 | 0.885 | Beta |
| Unstable knee after conservative treatment | 0.410 | 0.205 | 0.615 | Beta |
| Meniscus surgery after ACLR | 0.007 | 0.003 | 0.010 | Beta |
| Other surgery after ACLR | 0.003 | 0.001 | 0.004 | Beta |
| ACLR revision after primary ACLR | 0.010 | 0.005 | 0.015 | Beta |
| Meniscus surgery after conservative treatment alone | 0.006 | 0.003 | 0.009 | Beta |
| Delayed ACLR | 0.385 | 0.192 | 0.577 | Beta |
| Meniscus surgery after delayed ACLR | 0.010 | 0.005 | 0.015 | Beta |
| Others surgery after delayed ACLR | 0.006 | 0.003 | 0.009 | Beta |
| ACLR revision after delayed ACLR | 0.006 | 0.003 | 0.009 | Beta |
| Conservative with further meniscus surgery | 0.006 | 0.003 | 0.009 | Beta |
| **Utility (quality of life)** |  |  |  |  |
| Stable knee after ACLR | 0.86 | 0.61 | 1 | Beta |
| Stable knee after conservative treatment | 0.81 | 0.61 | 1 | Beta |
| Stable knee after delayed ACLR | 0.81 | 0.61 | 1 | Beta |
| **Cost (US$)^a,b^** |  |  |  |  |
| ACLR surgery | 4192 | 2096 | 6288 | Gamma |
| Conservative treatment | 1496 | 748 | 2244 | Gamma |
| Meniscus surgery | 2286 | 1143 | 3429 | Gamma |
| Other surgery | 1325 | 663 | 1988 | Gamma |
| Delayed ACLR surgery | 5688 | 2844 | 8532 | Gamma |
| Indirect cost: loss of salary (1 month) | 50 | 25 | 75 | Gamma |
| Indirect cost: transportation cost for 1 meeting | 3.3 | 1.7 | 5 | Gamma |
| Medical consultation (1 session) | 13.24 | 6.62 | 19.86 | Gamma |
| **Miscellaneous** |  |  |  |  |
| Discount, rate, % | 3 | 1 | 5 | Beta |

ACLR, anterior cruciate ligament reconstruction surgery.

^a^ Lower-value costs are based on payment of national insurance of Indonesia (BPJS) without indirect costs.

^b^ Upper-value costs are 150% of the base cost value.

^#^ Base case value is the weighted average from referenced studies. The lower and upper values for probabilities, rates, health utilities, and

costs are 50% and 150% of the base case value, respectively.

**Sensitivity Analysis: Eff-aclr**

**Early ACLR Surgery vs. Conservative**


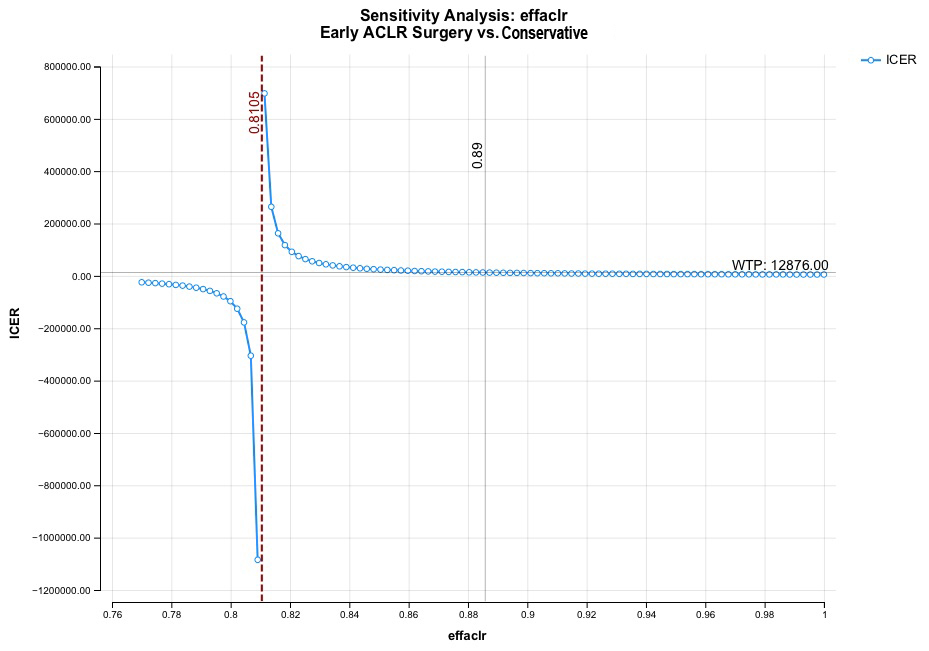


Supplementary Fig 1. One-way sensitivity analysis for quality of life of the early ACLR group showed that the quality of life of ACL injury patients after ACLR treatment should be more than 0.89 to be a more cost-effective strategy than conservative treatment. Eff-aclr: effectiveness of ACLR. ICER, incremental cost-effectiveness ratio. WTP: willingness-to-pay.
